# Antimicrobial and Antibiofilm Activities of Glycyl–Histidine and Methionyl–Glycine Dipeptides: In Vitro and Molecular Docking Studies

**DOI:** 10.3390/molecules31101641

**Published:** 2026-05-13

**Authors:** Gulcan Sahal, Tuğçe Deniz Karaca, Yusuf Sert, Meltem Maras, Alev Doğan

**Affiliations:** 1 Department of Biology, Biotechnology Division, Faculty of Science, Hacettepe University, 06800 Ankara, Türkiye; 2 Department of Medical Services and Techniques, Vocational School of Health Services, Gazi University, 06830 Ankara, Türkiye; tdenizkaraca@gazi.edu.tr; 3 Department of Physics, Faculty of Art & Sciences, Yozgat Bozok University, 66900 Yozgat, Türkiye; yusuf.sert@bozok.edu.tr; 4 Department of Mathematics and Science Education, Faculty of Eregli Education, Zonguldak Bülent Ecevit University, 67300 Zonguldak, Türkiye; melmaras@yahoo.com.tr; 5 Department of Science Education, Faculty of Gazi Education, Gazi University, 06500 Ankara, Türkiye; alevd@gazi.edu.tr

**Keywords:** dipeptides, antimicrobial activity, antibiofilm activity, ESKAPE pathogens, *C. albicans*, molecular docking, glycyl–histidine

## Abstract

The increasing prevalence of antimicrobial resistance and biofilm-associated infections has intensified the search for alternative anti-infective strategies. Short peptide-based molecules have attracted growing interest due to their structural simplicity, biocompatibility, and multifunctional biological properties. In this study, the antimicrobial and antibiofilm activities of two dipeptides, glycyl–histidine and methionyl–glycine, were evaluated against reference microorganisms, including *Escherichia coli* ATCC 35218, *Klebsiella pneumoniae* MTCC 109, *Pseudomonas aeruginosa* ATCC 27853, *Staphylococcus aureus* ATCC 25923, and *Candida albicans* ATCC 10231. Antimicrobial activity was determined using broth microdilution and disk diffusion assays, while antibiofilm effects were evaluated at sub-inhibitory concentrations using a crystal violet-based biofilm inhibition assay supported by light microscopy. In addition, the electronic structure, binding interactions, and pharmacokinetic properties of the dipeptides were investigated using integrated density functional theory (DFT), molecular docking, and ADME analyses. Glycyl–histidine exhibited antimicrobial activity against all tested bacterial strains (MIC: 12.5 mg/mL) and against *C. albicans* (MIC: 50 mg/mL), whereas methionyl–glycine showed no detectable antimicrobial activity. Both dipeptides demonstrated microorganism-dependent antibiofilm effects, with glycyl–histidine consistently displaying stronger activity. Notably, glycyl–histidine reduced biofilm formation by up to 88% in *K. pneumoniae* and by 54% in *P. aeruginosa* at 0.5 × MIC. In *C. albicans*, biofilm formation decreased by 22–39% under conditions where the reference antibiotic solution showed no antibiofilm effect. Computational analyses supported the experimental findings and provided molecular-level insights into the antimicrobial and antibiofilm potential of glycyl–histidine. Overall, these results identify glycyl–histidine as a promising anti-infective dipeptide and highlight its potential as a promising building block for the development of novel anti-infective agents.

## 1. Introduction

The emergence of antimicrobial resistance, together with the increasing prevalence of biofilm-associated infections, has become a critical global health challenge, significantly limiting the effectiveness of conventional antimicrobial therapies [1]. Because microbial biofilms exhibit enhanced tolerance to antimicrobial agents and host immune responses, they are responsible for persistent infections, increased morbidity, and higher treatment costs [2,3]. Additionally, ESKAPE pathogens (*Enterococcus faecium*, *Staphylococcus aureus*, *Klebsiella pneumoniae*, *Acinetobacter baumannii*, *Pseudomonas aeruginosa*, and *Enterobacter* spp.), which account for the majority of multidrug-resistant and biofilm-associated infections, are increasingly able to “escape” the effects of conventional antimicrobial therapies [4,5]. Therefore, identifying alternative or complementary antimicrobial compounds capable of inhibiting both planktonic growth and biofilm formation remains highly important.

Recently, the development of peptide-based compounds as therapeutic agents has emerged as a promising strategy in the fight against antimicrobial resistance [6,7]. Peptides are frequently cited in the literature as preferred compounds, particularly for targeting complex and challenging targets such as protein complexes, due to their high specificity and innovative strategies. Furthermore, natural antimicrobial peptides (AMPs) have been identified to date, and many improvements have been achieved through specific chemical modifications of these peptides [8]. AMPs are generally short peptide chains with a broad spectrum of activity against microorganisms [9]. Many also exhibit antibiofilm activity, preventing biofilm formation and adhesion [10,11,12]. However, when research on peptides is examined, it is seen that research on dipeptides is relatively limited. Dipeptides are small peptides that possess characteristics such as solubility, high affinity, strong specificity, low toxicity, and membrane interaction ability. Small peptides are often considered model compounds and can be easily targeted to biomolecules. Therefore, research on dipeptides is also important in contributing to this field [13,14,15,16,17].

Glycyl–histidine and methionyl–glycine are two distinct low-molecular-weight dipeptides composed of different amino acids but sharing a common glycine residue, which is the smallest nonessential amino acid in humans and animals and characterized by the absence of a side chain and high conformational flexibility [18,19]. This property facilitates close packing within various peptide structures [19]. Owing to these characteristics, glycine-containing peptides can readily adapt their conformation to interact with biological membranes and macromolecular targets. In line with this, glycine has been reported to exhibit antimicrobial and antibiofilm activities under in vitro conditions, supporting the biological relevance of glycine-containing compounds in the development of alternative antimicrobial strategies [20]. Among these dipeptides, glycyl–histidine is a histidine-containing dipeptide that has attracted attention due to the diverse biological functions of histidine, which arise from the structural versatility of its side chain, particularly the imidazole ring [21,22]. On the other hand, methionyl–glycine is a methionine-containing dipeptide characterized by the presence of a highly hydrophobic amino acid [23] and its distinctive sulfur-containing side chain [24]. These properties contribute to redox-related biochemical processes and hydrophobic interactions [23,24].

Therefore, in this study, the antimicrobial and antibiofilm potentials of glycyl–histidine and methionyl–glycine dipeptides were investigated against five clinically relevant reference strains, including *Escherichia coli* ATCC 35218, *Klebsiella pneumoniae* MTCC 109, *Staphylococcus aureus* ATCC 25923, and *Pseudomonas aeruginosa* ATCC 27853, which are closely associated with the ESKAPE group of pathogens, as well as *Candida albicans* ATCC 10231, a clinically relevant biofilm-forming opportunistic fungal pathogen. In addition, comprehensive in silico ADMET and drug similarity analyses were performed to evaluate the pharmacokinetic behavior, safety profile, and oral bioavailability potential of these peptides. The findings of this study provide new insight and will contribute to the advancement of new strategies involving dipeptide-based synthesizable molecules for antimicrobial and antibiofilm applications.

## 2. Results and Discussion

The antimicrobial and antibiofilm potential of the investigated dipeptides, glycyl–histidine and methionyl–glycine, was evaluated using antimicrobial susceptibility assays against clinically relevant bacterial and fungal reference strains. In addition, in silico analyses were performed to provide further insight into their biological activity and potential mechanisms of action.

### 2.1. MICs and MMCs of Glycyl–Histidine and Methionyl–Glycine Dipeptides

The MIC and MMC analyses demonstrated that glycyl–histidine was the only dipeptide exhibiting antimicrobial activity against all tested microorganisms, including the Gram-negative bacteria *E. coli*, *K. pneumoniae*, and *P. aeruginosa*, the Gram-positive bacterium *S. aureus*, and the fungal strain *C. albicans* (Table 1). Glycyl–histidine showed MIC values of 12.5 mg/mL against all bacterial strains and 50 mg/mL against *C. albicans*. In contrast, methionyl–glycine did not exhibit detectable antimicrobial activity at the highest tested concentrations (Table 1). The penicillin–streptomycin–amphotericin B solution used as the reference control showed strong antimicrobial activity against all tested bacterial strains, with MIC values ranging from 1.95 to 62.5 µL/mL, corresponding to approximately 39–625 µg of streptomycin, 0.098–1.6 µg of amphotericin B, and 39–625 U of penicillin G (≈0.024–0.39 mg/mL) (Table 1). As expected, glycyl–histidine required higher concentrations to inhibit microbial growth compared with the antibiotic control. Although relatively high inhibitory concentrations were observed, this behavior is not uncommon for short peptide-based compounds. Short peptides are generally associated with favorable biocompatibility and low toxicity profiles [25] and glycine-containing peptides have been reported to exhibit low cytotoxicity toward mammalian cells [26]. In addition, peptide-based molecules may exert beneficial host-related effects, such as modulation of inflammatory responses and promotion of wound healing [4,27,28]. However, such literature-based findings do not substitute for compound-specific cytotoxicity evaluation, particularly at the relatively high concentrations required for antimicrobial activity, and the absence of such analysis represents a limitation of the present study.

### 2.2. ZOIs of Glycyl–Histidine and Methionyl–Glycine Dipeptides

**Figure 1:**
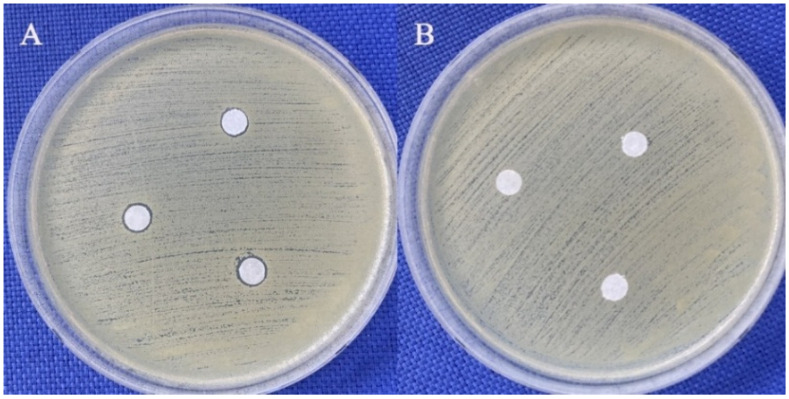
Agar diffusion assay images demonstrating the antimicrobial effects of dipeptides against *S. aureus* ATCC 25923. (**A**) Glycine-histidine, showing a visible inhibition zone. (**B**) Methionyl–glycine, showing no inhibition zone. In both assays, 5 µL of the respective dipeptide solution (200 mg/mL) was applied to each of three disks.

According to the disk diffusion assay results, 200 mg/mL glycyl–histidine produced inhibition zones against all tested bacterial microorganisms, whereas no inhibition zone was observed for *C. albicans*. In contrast, 200 mg/mL methionyl–glycine did not produce any inhibition zones against any of the tested microorganisms (Table 2 and Figure 1). The reference antibiotic disks generated distinct inhibition zones against each tested bacterial strain (Table 2).

These findings are consistent with the MIC/MMC results and confirm the antimicrobial activity of glycyl–histidine against planktonic bacterial cells under diffusion-based conditions. The absence of inhibition against *C. albicans* is also consistent with the MIC/MMC data, which showed a higher MIC value for this strain than for the bacterial strains, indicating lower susceptibility of fungal cells to glycyl–histidine under the tested conditions [29]. This reduced effectiveness may be attributed to structural differences in fungal cell envelopes, which can limit peptide accessibility to the membrane.

In addition, differences in membrane composition, such as the presence of ergosterol in fungal membranes, may further reduce susceptibility to peptide-mediated antimicrobial effects [30]. In contrast to glycyl–histidine, methionyl–glycine did not exhibit measurable antimicrobial activity in either the MIC/MMC or disk diffusion assays against any of the tested microorganisms (Table 1 and Table 2; Figure 1).

This lack of activity suggests that the presence of the hydrophobic sulfur-containing methionine residue [23] alone is insufficient to confer antimicrobial efficacy. By comparison, glycyl–histidine contains a histidine residue with an imidazole side chain [21] that can acquire a positive charge under physiological conditions, providing a cationic character important for antimicrobial activity [22]. The cationic imidazole ring facilitates electrostatic interactions with negatively charged microbial cell surfaces, promoting membrane association and destabilization, which are key mechanisms underlying the activity of many peptide-based antimicrobial compounds [31]. Accordingly, glycyl–histidine may interact more effectively with the microbial membrane surface via electrostatic interactions and accumulate by associating with lipid head groups. In general, it can be said that there is a bacteriostatic effect here, resulting from a combination of electrostatic interaction at the membrane surface + diffusion/porin passage + low-affinity binding to intracellular targets. These effects are inconsistent with the Barrel-stave model and Toroidal pore model, which are among the proposed mechanisms for peptides in the literature. Due to its limited hydrophobicity, glycyl–histidine is unlikely to deeply penetrate the membrane; instead, it may form a surface-associated layer that disrupts membrane charge balance and lipid organization, leading to increased permeability [32].

The results obtained show that the mechanism of action of glycyl–histidine peptide coincides with the “carpet model” described in the literature and leads to instability in the cell membrane. In this case, the compound accumulates on the bacterial membrane surface due to electrostatic attraction. This accumulation disrupts the structural integrity of the membrane and leads to the formation of mycelium-like structures. As a result, cellular contents leak into the external environment, and cellular damage occurs [33].

### 2.3. Biofilm Inhibitory Effects of Glycyl–Histidine and Methionyl–Glycine Dipeptides

Antibiofilm activity is a critical determinant of clinical relevance. Biofilm formation enhances microbial tolerance to antimicrobial agents and host immune defenses [3,34]. This mode of growth markedly enhances microbial tolerance to antimicrobial agents and reduces susceptibility to host immune defenses [35]. Therefore, the antibiofilm potential of glycyl–histidine and methionyl–glycine against reference bacterial and fungal strains was evaluated in the present study.

**Figure 2:**
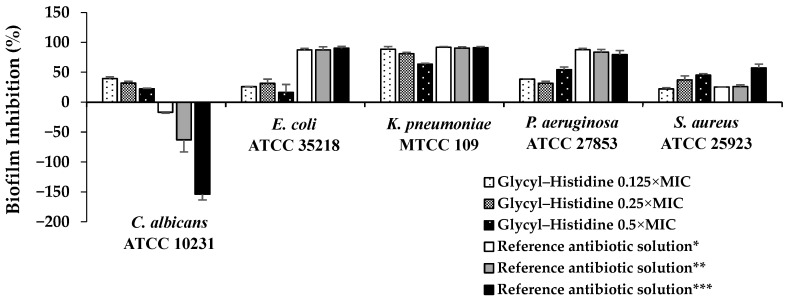
Biofilm inhibitory effects (%) of glycyl–histidine at sub-MICs against *C. albicans* ATCC 10231, *E. coli* ATCC 35218, *K. pneumoniae* MTCC 109, *P. aeruginosa* ATCC 27853, and *S. aureus* ATCC 25923. For bacterial strains, the reference antibiotic solution (penicillin–streptomycin–amphotericin B) was tested at the same fractional MIC values (* 0.125 × MIC, ** 0.25 × MIC, *** 0.5 × MIC). For *C. albicans*, as the MIC of the reference antibiotic solution was > 500 mg/mL, concentrations of 125, 250, and 500 mg/mL were tested (*, **, and ***, respectively). Negative values indicate biofilm enhancement relative to the untreated control. Error bars represent standard deviations of three independent experiments.

**Figure 3:**
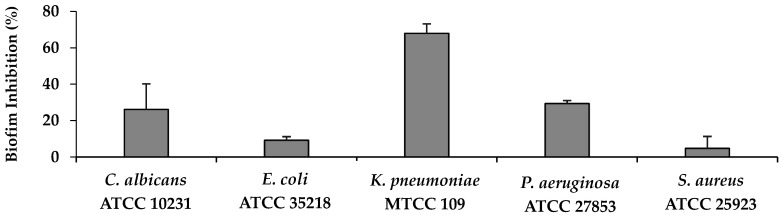
Biofilm inhibitory effects (%) of methionyl–glycine at 12.5 mg/mL. Error bars represent standard deviations of three independent experiments.

**Figure 4:**
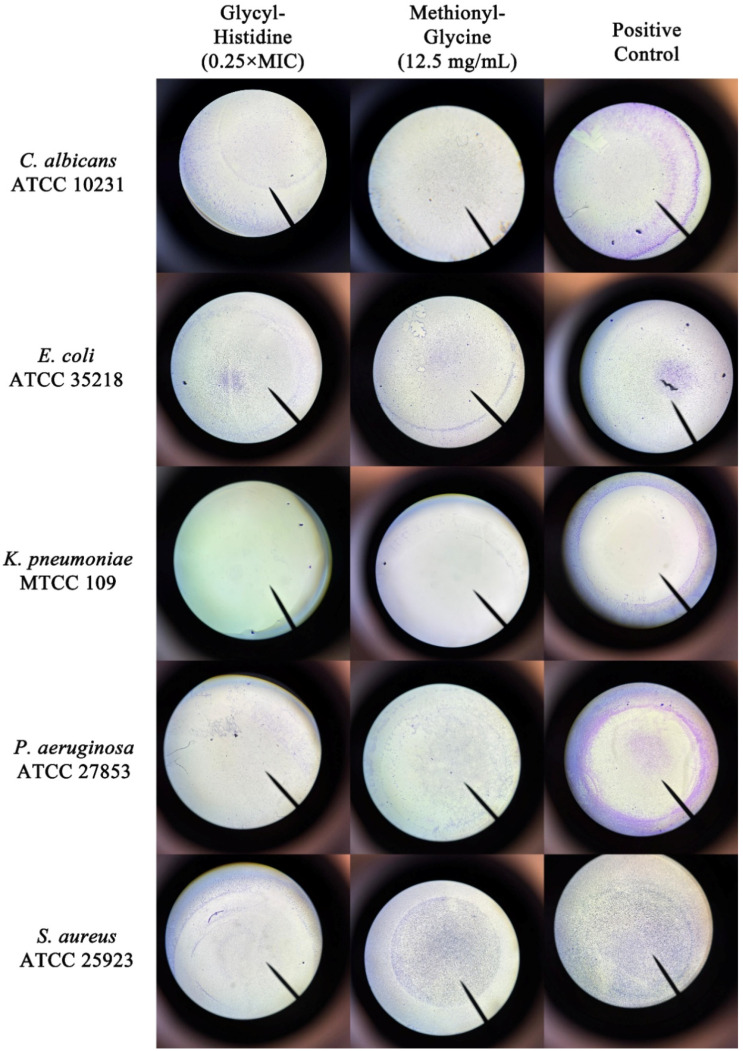
Light microscopy images (4× magnification) illustrating the effects of glycyl–histidine and methionyl–glycine on biofilm formation by *C. albicans* ATCC 10231, *E. coli* ATCC 35218, *K. pneumoniae* MTCC 109, *P. aeruginosa* ATCC 27853, and *S. aureus* ATCC 25923. For glycyl–histidine, images corresponding to 0.25 × MIC were used for all tested microorganisms. For methionyl–glycine, biofilm images were obtained at a concentration of 12.5 mg/mL for all microorganisms. Positive control images represent biofilm formation in the absence of any dipeptide under identical experimental conditions. Images are representative of three independent experiments.

As shown in Figure 2, glycyl–histidine exhibited limited biofilm inhibition against *E. coli* at sub-MIC levels, with inhibition percentages ranging between 20% and 40%, and no clear dose–response relationship was observed. In contrast, biofilm formation in *K. pneumoniae* was strongly inhibited by glycyl–histidine at 0.125 × MIC and 0.25 × MIC, reaching inhibition levels of approximately 80–90%. Similarly, methionyl–glycine also demonstrated its strongest antibiofilm effect against *K. pneumoniae*, achieving 68% inhibition (Figure 3 and Figure 4). The strong susceptibility of *K. pneumoniae* may be related to the structural features governing biofilm formation in this organism. In *K. pneumoniae*, lipopolysaccharides (LPS) and the capsular polysaccharide play interconnected roles in biofilm formation, with LPS contributing primarily to the initial adhesion to abiotic surfaces, while the capsule is essential for effective surface coverage and the establishment of mature biofilm architecture [36]. The small dipeptides evaluated in this study may therefore have interfered with the functional roles of these surface-associated components during early adhesion and extracellular matrix assembly, thereby impairing biofilm development. *Klebsiella pneumoniae* is a Gram-negative enteric pathogen frequently associated with healthcare-related infections, particularly in intensive care units, where it is implicated in ventilator-associated pneumonia, bloodstream infections, catheter-associated infections, and urinary tract infections [37,38]. Its ability to form strong biofilms on medical devices and abiotic surfaces significantly contributes to bacterial persistence, increased antimicrobial tolerance, and treatment failure [36,39]. Consequently, biofilm-associated *K. pneumoniae* infections are often difficult to eradicate and may require prolonged or combination antimicrobial therapies [40]. Therefore, the high levels of biofilm inhibition observed for both dipeptides in this study are of particular clinical relevance. For *P. aeruginosa*, glycyl–histidine exhibited a concentration-dependent antibiofilm effect, with the highest inhibition observed at 0.5 × MIC (54% inhibition) (Figure 2 and Figure 4), whereas methionyl–glycine showed only moderate inhibition (29%) (Figure 3 and Figure 4). Biofilm formation in *P. aeruginosa* is tightly regulated by quorum sensing-dependent signaling networks that coordinate extracellular matrix production and biofilm maturation [41]. In addition, lipopolysaccharides contribute to surface attachment, while exopolysaccharides such as Psl and Pel play key roles in biofilm establishment and structural stability [42]. Although quorum sensing is an important regulatory mechanism, the pronounced antibiofilm activity of glycyl–histidine observed in both *K. pneumoniae* and *P. aeruginosa*—organisms characterized by LPS- and polysaccharide-dependent biofilm formation—suggests that the observed effects may be more closely associated with interference in surface-associated or matrix-related processes rather than direct modulation of quorum sensing pathways. In contrast to the relatively strong antibiofilm effects observed for *K. pneumoniae* and *P. aeruginosa*, both glycyl–histidine and methionyl–glycine exhibited limited antibiofilm activity against *E. coli* (Figure 2, Figure 3 and Figure 4). Biofilm formation in *E. coli* is primarily mediated by adhesion-associated surface structures such as flagella, fimbriae, and curli fibers [43]. This limited response suggests a reduced ability of the dipeptides to interfere with adhesion-driven biofilm formation mechanisms in *E. coli*. When evaluated against *S. aureus*, glycyl–histidine demonstrated a moderate antibiofilm effect, reaching 45% inhibition at 0.5 × MIC (Figure 2), whereas methionyl–glycine showed only minimal antibiofilm activity (5% inhibition) (Figure 3 and Figure 4). Biofilm formation in *S. aureus* involves multiple surface-associated components, including polysaccharide intercellular adhesin (PIA), teichoic acids, and extracellular DNA, all of which contribute to biofilm development and structural stability [44]. The antibiofilm activity of glycyl–histidine may therefore be associated with interactions between its imidazole-containing histidine residue and these surface- or matrix-associated components. For *C. albicans*, glycyl–histidine exhibited a moderate antibiofilm effect at sub-MICs, resulting in approximately 20–30% biofilm inhibition, with the highest inhibition reaching 39% at 0.125 × MIC (6.25 mg/mL) (Figure 2 and Figure 4). In comparison, methionyl–glycine showed only limited antibiofilm activity, resulting in approximately 26% inhibition at 12.5 mg/mL (Figure 3 and Figure 4). Notably, despite being tested at a lower relative concentration, glycyl–histidine produced a stronger antibiofilm effect than methionyl–glycine, suggesting that antibiofilm efficacy against *C. albicans* is strongly influenced by dipeptide-specific chemical properties. The presence of the ionizable imidazole ring in glycyl–histidine may facilitate interactions with charged or polar components of the fungal cell surface or extracellular matrix, whereas the relatively hydrophobic nature of methionyl–glycine may limit its ability to effectively interfere with fungal biofilm development.

Biofilm formation in *C. albicans* is a complex multistep process involving initial adhesion to surfaces, morphological transitions from yeast to hyphal forms, and production of a dense extracellular matrix composed of polysaccharides, proteins, and lipids, all of which contribute to biofilm stability and increased antifungal tolerance [45]. Interestingly, the reference antibiotic solution increased *C. albicans* biofilm formation, whereas glycyl–histidine reduced biofilm formation under the same conditions, indicating a distinct mode of action.

### 2.4. DFT and Molecular Docking

Density Functional Theory (DFT) calculations play a fundamental role in modern computational chemistry by providing reliable information about the intrinsic electronic structure, geometric stability, and physicochemical properties of bioactive molecules. Unlike empirical methods, DFT offers a quantum mechanical description of molecular systems, enabling accurate prediction of optimized geometries, electronic energies, dipole moments, and reactivity-related descriptors. In structure-based drug design studies, the reliability of molecular docking results strongly depends on the accuracy of ligand geometries. Therefore, prior quantum mechanical optimization ensures that ligands are evaluated in their most stable conformational states, minimizing artificial strain effects during docking simulations. In this context, DFT serves as the theoretical foundation that supports subsequent molecular recognition analyses [46,47,48,49,50,51,52,53,54,55,56,57,58,59,60].

**Figure 5:**
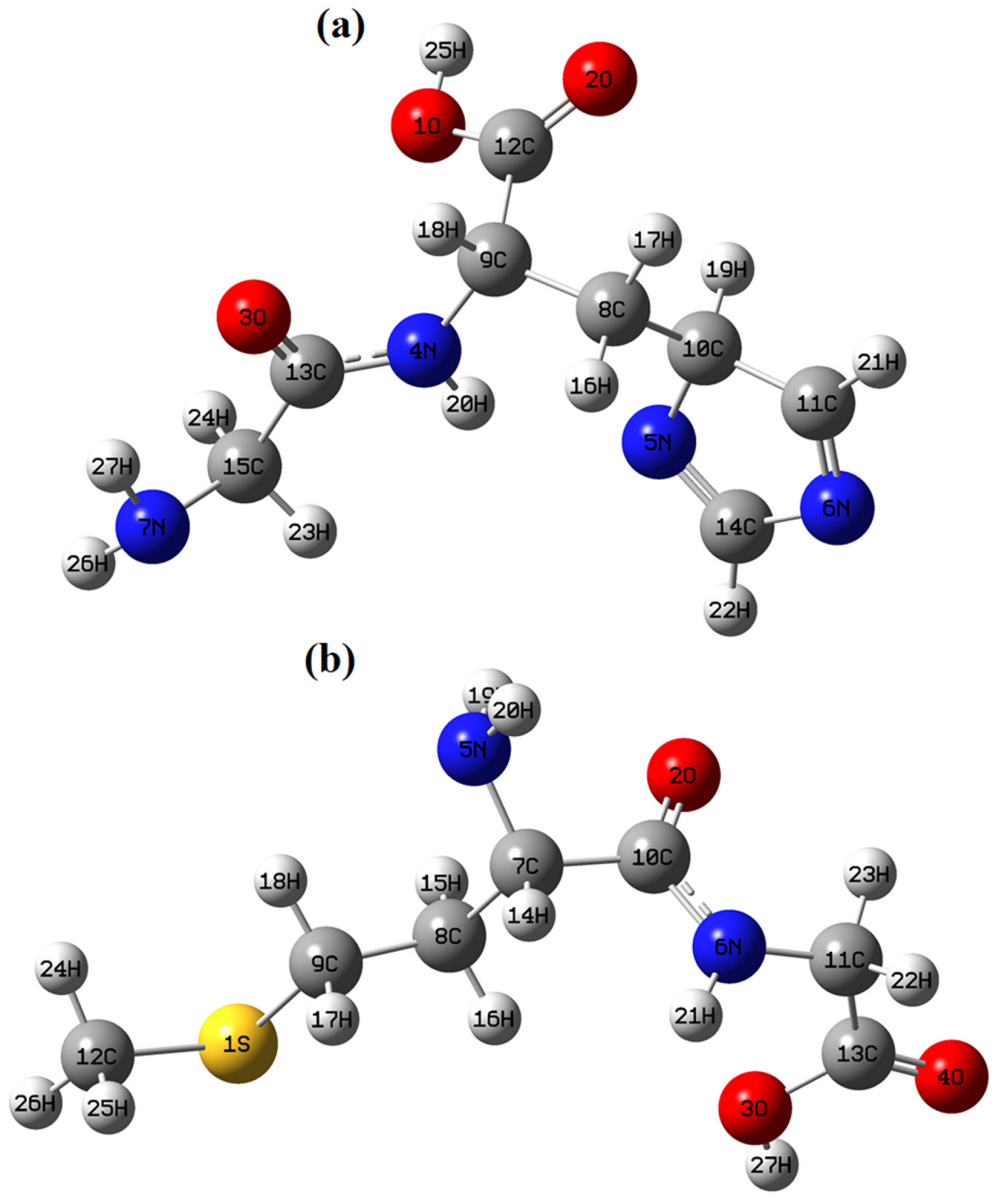
The optimized structures of glycyl–histidine (**a**) and methionyl–glycine (**b**) molecules.

Molecular docking, on the other hand, represents a powerful in silico technique for predicting the interaction pattern between small molecules and biological macromolecules. It provides insights into binding affinity, orientation within the active site, and stabilizing non-covalent interactions such as hydrogen bonding, hydrophobic contacts, van der Waals forces, and electrostatic complementarity. When combined with DFT-based structural optimization, docking simulations yield a more robust and theoretically consistent evaluation platform. This integrated computational approach enables the estimation of theoretical inhibitory potentials prior to experimental validation and significantly reduces time and resource consumption in drug discovery processes [61,62,63,64,65]. In silico methods such as DFT and molecular docking provide valuable molecular-level insights [6]; however, they do not substitute for experimental validation. Therefore, the computational findings in this study should be interpreted as complementary to the experimental results rather than as standalone evidence of biological activity. In the present study, the theoretical inhibitory potentials of glycyl–histidine (GLYH) and methionyl–glycine (MGLY) were investigated against antibiofilm (PDB: 2UVO) and antimicrobial (PDB: 8BN6) targets through molecular docking analysis. These biological targets were selected based on prior experimental evidence and their reported involvement in bacterial regulatory pathways related to virulence and biofilm formation. Specifically, the proteins corresponding to PDB IDs 2UVO and 8BN6 are known to participate in key biological processes such as quorum sensing, metabolic regulation, and cellular persistence, which are critical for microbial survival and biofilm development. Therefore, evaluating the interactions between the studied compounds and these targets provides a mechanistic framework that supports the experimentally observed antimicrobial and antibiofilm effects. It should be emphasized that the docking analysis does not aim to establish definitive mechanisms, but rather to provide supportive molecular-level insights into the observed biological activity. Initially, glycyl–histidine and methionyl–glycine molecules were fully optimized at the DFT level using the Gaussian 09W package program [66] and GaussView 5.0 interface [67]. Calculations were performed in the gas phase employing the DFT functional with the 6-311++G(d,p) basis set [68,69]. The optimized three-dimensional geometries are presented in Figure 5 (a: GLYH, b: MGLY). The calculated sum of electronic and zero-point energies and dipole moments was obtained as:

- Glycyl–histidine
  E = −756.97951311 a.u
  Dipole moment = 2.5160 Debye
- Methionyl–glycine
  E = −1008.78700931 a.u
  Dipole moment = 4.1802 Debye

The higher dipole moment of methionyl–glycine suggests relatively greater molecular polarity compared to glycyl–histidine, which may influence electrostatic complementarity within protein active sites. The optimized geometries were exported in PDB format and used as input structures for docking simulations. Ligand torsional flexibility was carefully examined using Discovery Studio [70], and ten conformational modes were generated for each ligand to ensure adequate conformational sampling. The crystal structures of 2UVO [71] and 8BN6 [72] proteins were retrieved from the RCSB Protein Data Bank [73]. Structural preparation, including removal of water molecules and cofactor structures as well as necessary corrections, was performed using Discovery Studio [70]. For the antibiofilm target 2UVO, although chains E, F, G, and H are present in the crystal structure [71], only chain E was considered during docking calculations. The active site residues were defined as TYR93, TRP88, CYR79, GLY126, ALA50, TRP60, TYR64, ASP73, THR75, SER129, TYR56, LEU110, and ALA105. The grid box parameters encompassing these residues were set as: center_x = 23.755, center_y = 13.502, center_z = 80.496, size_x = 74, size_y = 66, size_z = 80. For the antimicrobial target 8BN6 [72], since a predefined active residue list was not specified, the grid box was centered to include the cofactor binding region to ensure optimal binding energy acquisition. The grid parameters were defined as: center_x = −3.334, center_y = 123.781, center_z = 0.055, size_x = 40, size_y = 40, size_z = 40. Prior to docking calculations, redocking validation of cofactor structures was performed to confirm correct targeting of binding pockets. Molecular docking simulations were then conducted using AutoDock Vina [65]. The docking results for the first ten modes, including binding energies, RMSD values, hydrogen bond numbers, and calculated inhibition constants (Ki), are summarized in Table 3. According to Table 3, the strongest interaction for the 2UVO receptor was observed between glycyl–histidine and 2UVO with a binding energy of −6.8 kcal/mol.

**Figure 6:**
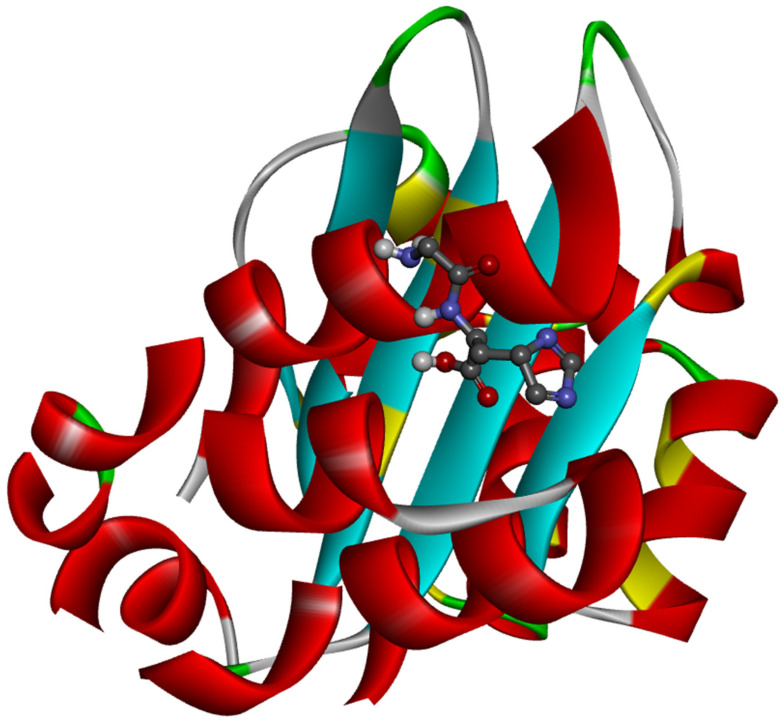
The placement of glycyl–histidine molecule within the active site of the 2UVO protein.

**Figure 7:**
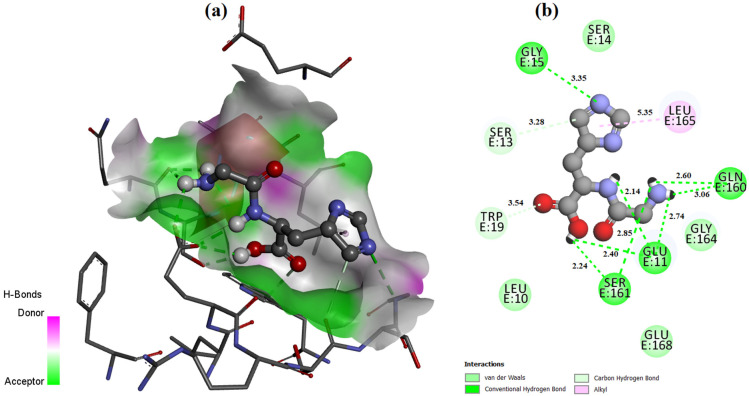
The 3D (**a**) and 2D (**b**) interaction representation between the glycyl–histidine molecule and PDB: 2UVO.

**Figure 8:**
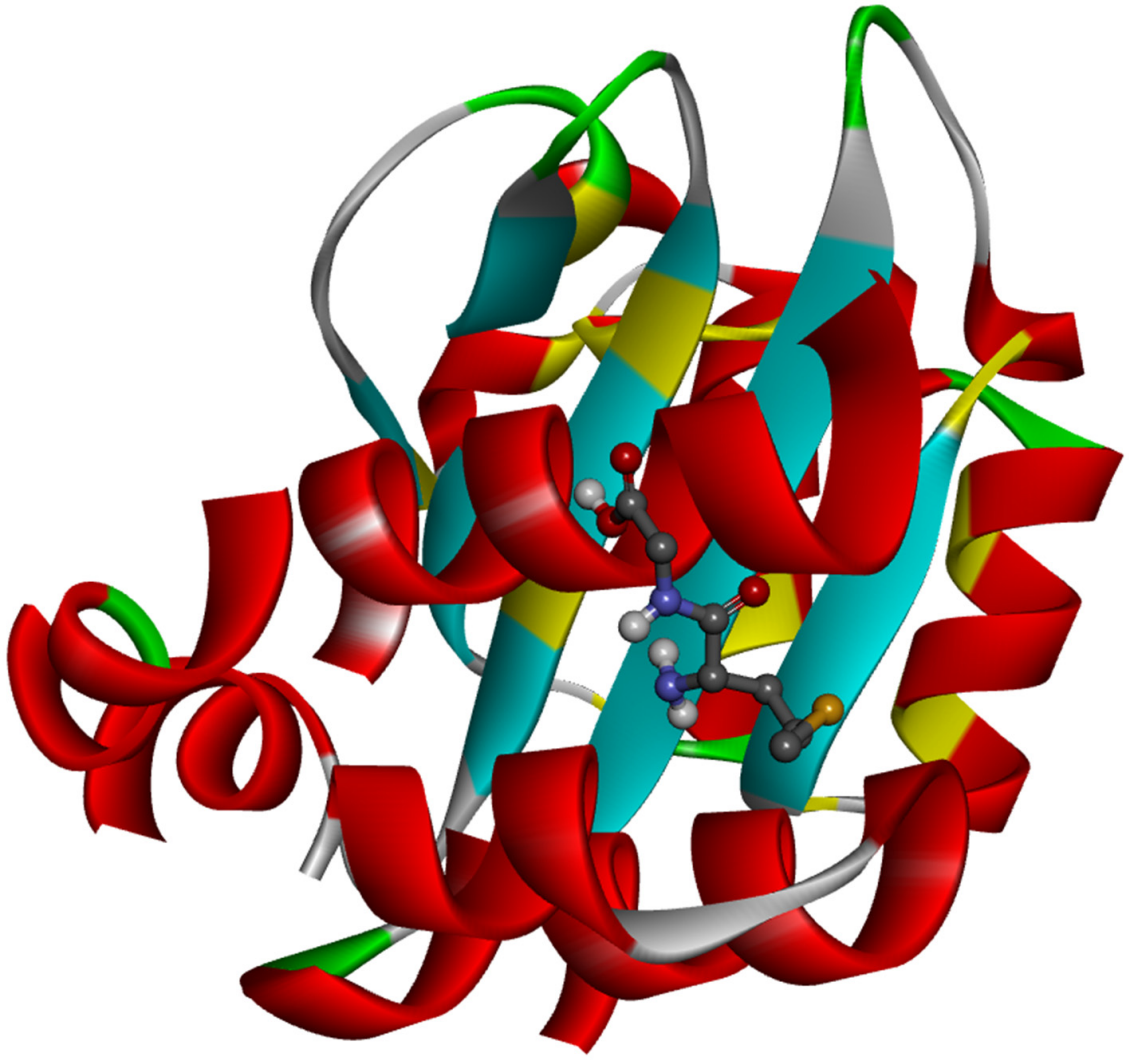
The placement of the methionyl–glycine molecule within the active site of the 2UVO protein.

**Figure 9:**
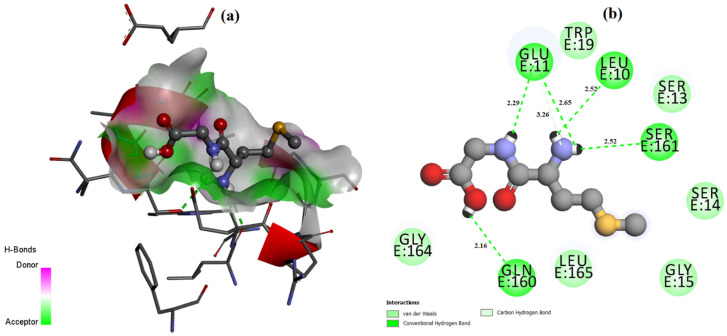
The 3D (**a**) and 2D (**b**) interaction representation between the methionyl–glycine molecule and PDB: 2UVO.

**Figure 10:**
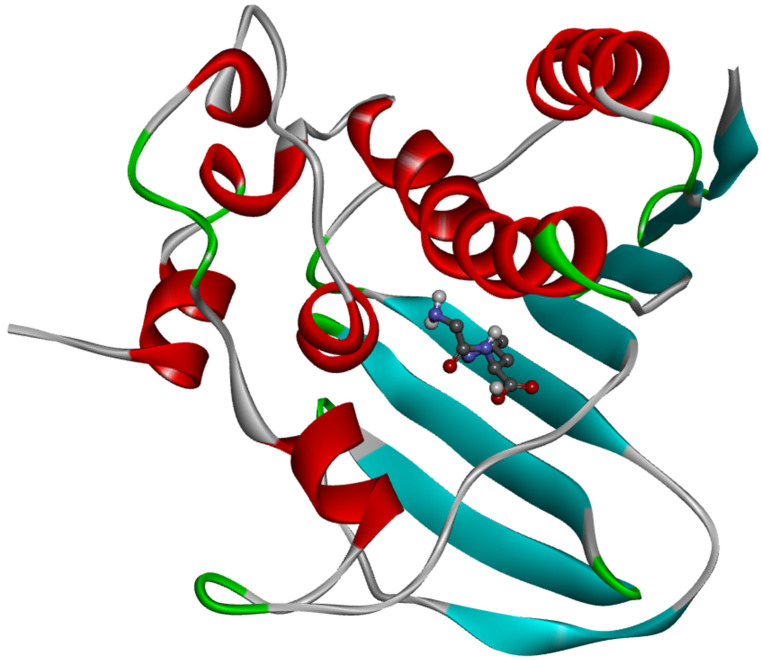
The placement of the glycyl–histidine molecule within the active site of the 8BN6 protein.

**Figure 11:**
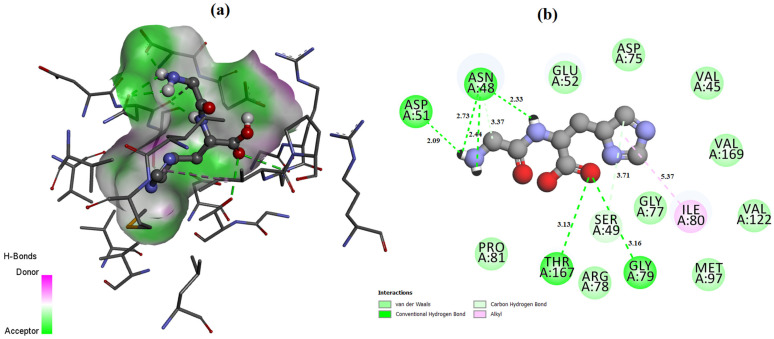
The 3D (**a**) and 2D (**b**) interaction representation between the glycyl–histidine molecule and PDB: 8BN6.

**Figure 12:**
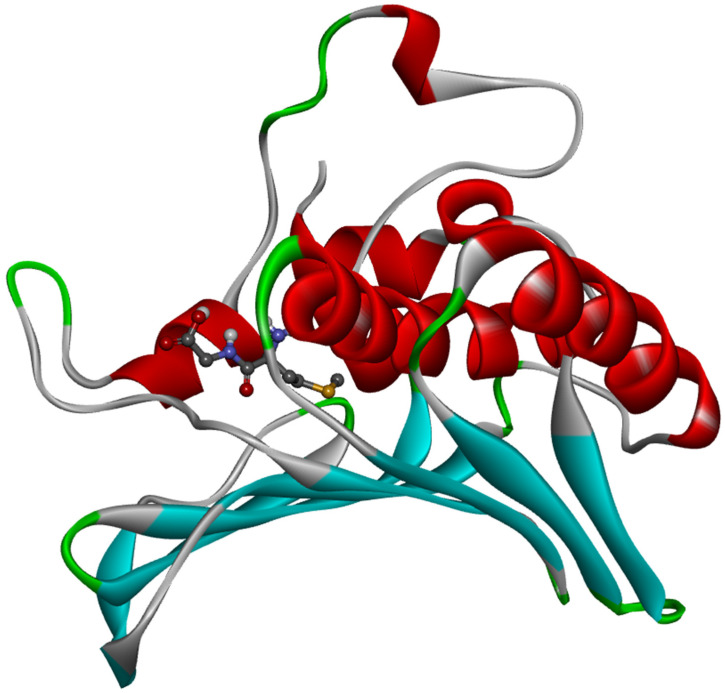
The placement of the methionyl–glycine molecule within the active site of the 8BN6 protein.

**Figure 13:**
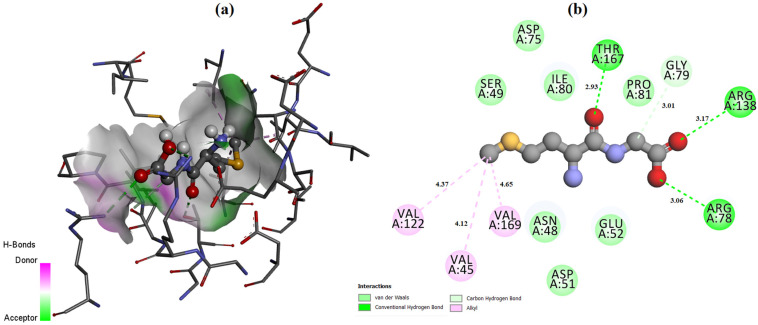
The 3D (**a**) and 2D (**b**) interaction representation between the methionyl–glycine molecule and PDB: 8BN6.

The ligand placement within the active site is illustrated in Figure 6, while the corresponding 3D (Figure 7a) and 2D (Figure 7b) interaction diagrams are shown in Figure 7. Glycyl–histidine formed 8 conventional hydrogen bonds, indicating strong stabilization within the active pocket. The calculated inhibition constant was Ki = 10.3649 μM, suggesting moderate inhibitory potential. In contrast, methionyl–glycine exhibited a binding energy of −6.4 kcal/mol toward 2UVO. The binding orientation is presented in Figure 8, and the interaction diagrams are given in Figure 9. Methionyl–glycine formed 5 conventional hydrogen bonds, and the calculated inhibition constant was Ki = 66.3544 μM. The comparatively higher Ki value suggests weaker antibiofilm inhibitory potential relative to glycyl–histidine. For the antimicrobial target 8BN6, the most favorable interaction was obtained between glycyl–histidine and 8BN6 with a binding energy of −5.7 kcal/mol. The ligand placement is depicted in Figure 10, and the 3D/2D interaction representations are shown in Figure 11. Glycyl–histidine formed 5 conventional hydrogen bonds, with a calculated inhibition constant of Ki = 20.3594 μM. Methionyl–glycine demonstrated a slightly stronger binding energy of −6.3 kcal/mol toward 8BN6. The binding conformation is illustrated in Figure 12, and detailed interactions are shown in Figure 13. Methionyl–glycine formed 3 conventional hydrogen bonds, with a calculated inhibition constant of Ki = 24.1027 μM.

Direct comparison of docking-derived binding energies with values reported in different studies should be approached with caution, as such comparisons may be affected by variations in docking protocols, scoring functions, search algorithms, grid parameters, and protein–ligand preparation procedures. These methodological differences can lead to significant variability in calculated binding affinities. Therefore, docking scores are more appropriately interpreted in a relative manner within the same computational framework rather than as absolute indicators of binding strength. In this context, the docking results presented in this study are used to support the experimental findings by providing qualitative insights into potential molecular interactions. It should be noted that a direct correlation between docking-derived binding energies and experimentally observed antimicrobial activity is not always expected. Antimicrobial effects are inherently multifactorial and depend on various factors, including membrane permeability, intracellular accumulation, and interactions with multiple biological targets. Therefore, the docking results presented in this study should be interpreted as qualitative indicators of potential molecular interactions rather than quantitative predictors of biological activity. In addition, the relatively small differences in binding energies among the tested compounds fall within the inherent limitations and error margins of docking methodologies. Thus, docking analysis should be considered as a hypothesis-generating approach rather than a definitive validation of the mechanism of action. In order to better relate the computational findings with the experimental observations, the antimicrobial and antibiofilm activities observed in this study may be associated with multiple mechanisms rather than a single specific target. In addition to potential interactions with regulatory proteins suggested by docking analysis, small peptides are known to exert antimicrobial effects through mechanisms such as disruption of bacterial membrane integrity, interference with intracellular metabolic processes, and modulation of quorum-sensing pathways. The biological activity observed for the dipeptides in this study is likely the result of a combination of these effects, consistent with the multifactorial nature of antimicrobial activity.

### 2.5. Drug Likeness and ADME Analyses

**Figure 14:**
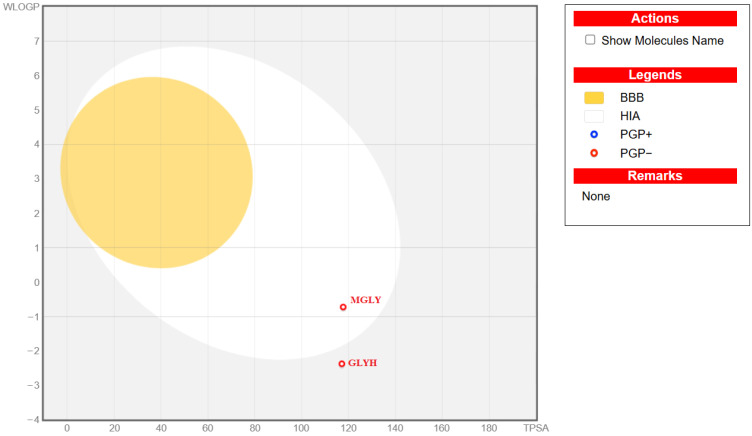
BOILED-EGG model representing the predicted gastrointestinal absorption (GI) and blood–brain barrier (BBB) permeability of glycyl–histidine (GLYH) and methionyl–glycine (MGLY). The model is based on lipophilicity (WLOGP, y-axis) and topological polar surface area (TPSA, Å^2^, x-axis). The white region indicates high probability of passive gastrointestinal absorption, while the yellow region (yolk) corresponds to molecules predicted to permeate the BBB.

**Figure 15:**
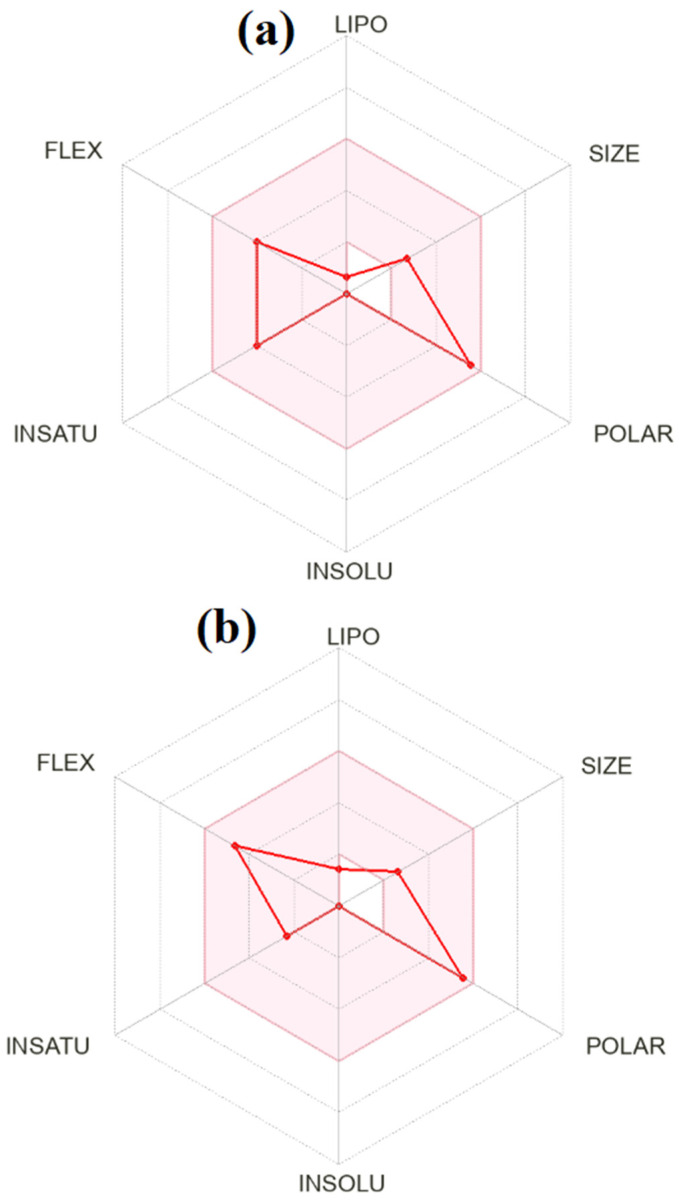
Bioavailability radar of (**a**) glycyl–histidine (GLYH) and (**b**) methionyl–glycine (MGLY), illustrating six physicochemical parameters relevant to oral bioavailability: lipophilicity (LIPO), molecular size (SIZE), polarity (POLAR, TPSA in Å^2^), solubility (INSOLU), flexibility (FLEX), and saturation (INSATU). The pink area represents the optimal range for oral bioavailability.

Drug-likeness and ADME evaluations are essential components of rational drug discovery, as strong binding affinity alone is insufficient for a molecule to become a viable therapeutic candidate. While molecular docking provides insight into target–ligand interactions at the molecular level, drug-likeness rules and pharmacokinetic predictions determine whether a compound possesses physicochemical and biological properties compatible with oral bioavailability and systemic activity [74,75,76,77,78]. Therefore, the drug-likeness and ADME profiles of glycyl–histidine and methionyl–glycine were predicted using the SwissADME web tool [74] based on their SMILES codes, and the calculated parameters were summarized in Table 4. The results were further interpreted using the BOILED-EGG model (Figure 14) and bioavailability radar plots (Figure 15). According to Table 4, both glycyl–histidine and methionyl–glycine fully comply with Lipinski’s Rule of Five [79], showing zero violations. Their molecular weights (212.21 and 206.26 g/mol) are well below the 500 g/mol threshold, and both compounds possess acceptable numbers of hydrogen bond donors and acceptors (GLYH: 3 HBD/6 HBA; MGLY: 3 HBD/4 HBA). The topological polar surface area (TPSA) values are approximately 117 Å^2^ for both molecules, remaining below the 140 Å^2^ limit generally associated with adequate intestinal absorption. Furthermore, both compounds satisfy Veber and Egan criteria, indicating favorable flexibility and polarity profiles for oral drug candidates. Lipophilicity analysis reveals that glycyl–histidine has a consensus LogP value of −1.71, whereas methionyl–glycine shows a less negative value of −0.84. This suggests that methionyl–glycine exhibits a more balanced hydrophilic–lipophilic profile compared to glycyl–histidine. Excessive hydrophilicity may limit passive membrane permeation, whereas excessive lipophilicity can reduce solubility. In agreement with these findings, SwissADME predicts low gastrointestinal (GI) absorption for glycyl–histidine but high GI absorption for methionyl–glycine. This difference is visually supported by the BOILED-EGG model (Figure 14), where methionyl–glycine is positioned within the region compatible with efficient intestinal absorption, while both compounds fall outside the blood–brain barrier (BBB) permeation zone.

The predicted lack of BBB permeability may be advantageous in minimizing central nervous system–related side effects. Water solubility predictions indicate that both molecules are classified as “highly soluble”. Glycyl–histidine exhibits higher predicted solubility values than methionyl–glycine, consistent with its more hydrophilic character. Although high solubility is favorable for dissolution and initial absorption, the lower lipophilicity of glycyl–histidine may contribute to its predicted lower GI absorption. In contrast, methionyl–glycine appears to present a more optimal balance between solubility and membrane permeability. Pharmacokinetic predictions further show that neither compound is expected to be a P-glycoprotein (P-gp) substrate, and neither is predicted to inhibit major cytochrome P450 isoenzymes (CYP1A2, CYP2C19, CYP2C9, CYP2D6, CYP3A4). This suggests a low potential for drug–drug interactions mediated through CYP inhibition. Additionally, both compounds exhibit very low skin permeation coefficients (Log Kp), indicating limited transdermal permeability. Importantly, PAINS and Brenk filters report zero alerts for both molecules, implying the absence of structural motifs associated with assay interference or reactive toxicophores [79]. The bioavailability radar plots presented in Figure 15 illustrate that both compounds largely fall within the optimal physicochemical space defined by lipophilicity, size, polarity, solubility, flexibility, and saturation. However, methionyl–glycine appears to occupy a more balanced region within the radar boundaries, particularly regarding lipophilicity and fraction Csp^3^ (0.71 for MGLY vs. 0.50 for GLYH), suggesting a more three-dimensional and saturated structure often associated with improved drug-likeness. When interpreted together with docking results, glycyl–histidine demonstrated stronger antibiofilm binding affinity, whereas methionyl–glycine displayed a more favorable pharmacokinetic profile, particularly in terms of predicted gastrointestinal absorption and lipophilicity balance. The BOILED-EGG model and bioavailability radar collectively support the conclusion that methionyl–glycine may have better oral drug potential, while glycyl–histidine exhibits strong target-binding capacity. Overall, both compounds satisfy fundamental drug-likeness criteria, show no structural toxicity alerts, and present acceptable ADME characteristics, supporting their potential as promising candidates for further experimental pharmacokinetic and in vivo validation.

Overall, the findings of this study demonstrate that dipeptides can exhibit selective and microorganism-dependent antimicrobial and antibiofilm effects that are strongly influenced by their amino acid composition. Despite sharing a common glycine residue, glycyl–histidine and methionyl–glycine displayed markedly different biological activities, highlighting the critical contribution of the histidine imidazole side chain to both antimicrobial and antibiofilm efficacy. The antibiofilm activity of glycyl–histidine, particularly against strong biofilm-forming microorganisms such as *K. pneumoniae* and *P. aeruginosa*, suggests that this simple dipeptide structure may serve as a promising building block for the development of more advanced antimicrobial strategies. Importantly, this work provides the first comprehensive and comparative insight into the antimicrobial and antibiofilm potentials of glycyl–histidine and methionyl–glycine against clinically relevant bacterial pathogens and the opportunistic fungal pathogen *C. albicans*.

Moreover, considering the computational studies, we can say the following: In this study, we employed an integrated DFT-based and structure-based computational approach to evaluate the antibiofilm and antimicrobial potentials of glycyl–histidine and methionyl–glycine. Quantum mechanical optimization ensured stable ligand conformations prior to docking analysis. Docking results indicated that glycyl–histidine exhibited stronger antibiofilm affinity toward 2UVO (−6.8 kcal/mol; Ki = 10.36 μM), whereas methionyl–glycine showed slightly better binding energy against the antimicrobial target 8BN6 (−6.3 kcal/mol). Low RMSD values confirmed docking reliability. Both compounds satisfied key drug-likeness criteria without structural alerts. While glycyl–histidine demonstrated superior target-binding capacity, methionyl–glycine displayed a more favorable pharmacokinetic profile, particularly in predicted gastrointestinal absorption. Overall, glycyl–histidine appears promising for antibiofilm applications, whereas methionyl–glycine offers advantageous pharmacokinetic characteristics, warranting further experimental validation.

It should be clearly acknowledged that the antimicrobial activity observed in this study is relatively weak, as indicated by the high MIC values. This limitation is consistent with the nature of short dipeptides, which often require higher concentrations to exert antimicrobial effects due to their limited structural complexity. However, dipeptide-based structures have increasingly been used in complexes with metallic nanoparticles, such as gold and silver, to enhance their biological activity. Such complexes generally exhibit a higher net positive charge than their non-complexed counterparts, enabling stronger electrostatic interactions with polyanionic biomolecules such as DNA and RNA. The bactericidal activity of such peptides can also be increased by complexing the metal-binding sites of the peptides with transition metals [80,81,82]. From a medical standpoint, bacitracin is a clinically important metal-dependent peptide antibiotic, whereas bleomycin is a metal-binding glycopeptide primarily used as an anticancer agent. These peptides exhibit their biological activities through specific interactions with cellular targets, including lipid carriers or DNA, depending on their structure and metal-complexing properties [83,84]. Taken together, these findings place the present results within a broader peptide-based antimicrobial research framework, while also highlighting important aspects that require further investigation. Within this broader context, in addition to the relatively weak antimicrobial activity observed, the lack of cytotoxicity analyses and the absence of stability, synergy, and polymicrobial biofilm evaluations represent the main limitations of the present work. Moreover, although well-characterized reference strains were used to ensure reproducibility, future studies should include a broader and more clinically relevant panel of microorganisms, including clinical and antibiotic-resistant isolates, particularly ESKAPE pathogens; more advanced imaging techniques may also provide additional structural insight.

## 3. Materials and Methods

### 3.1. Dipeptides

In this study, dipeptides, glycyl–histidine and methionyl–glycine (purity > 98%) were purchased from Ambeed Inc. (Ankara, Turkey) and used in the experiments. The molecular weights of glycyl–histidine and methionyl–glycine were 212.21 g/mol and 206.26 g/mol, respectively. Stock solutions of these dipeptides were prepared in 10 mM potassium phosphate-buffered solution (pH 7.0) and sterilized by UV irradiation for 1 h to be used in the tests.

### 3.2. Microorganisms

In this study, reference strains of *Candida albicans* ATCC 10231, *Escherichia coli* ATCC 35218, *Klebsiella pneumoniae* MTCC 109, *Pseudomonas aeruginosa* ATCC 27853 and *Staphylococcus aureus* ATCC 25923 were used. All microorganisms were cultured on Brain Heart Infusion (BHI) (Condalab, Madrid, Spain) agar and maintained as stock cultures at 4 °C.

### 3.3. Microbial Harvesting and Preparation of Microbial Suspensions

Single colonies of reference strains obtained from stock cultures were inoculated into 10 mL of BHI broth (pH 7.0) and incubated at 37 °C for 24 h. After incubation, 1.5 mL of each preculture was transferred into 30 mL of fresh BHI broth and incubated again under the same conditions for 24 h to obtain the main cultures. The main cultures were washed three times with 10 mM potassium phosphate buffer (pH 7.0) by centrifugation at 4000 rpm for 10 min. Following the washing steps, the resulting cell suspensions were adjusted to McFarland standard 2 turbidity.

### 3.4. Antimicrobial Tests (MIC, MMC and Disk Diffusion Assays)

Minimum Inhibitory Concentrations (MICs), Minimum Microbicidal Concentrations (MMCs), and Zone of Inhibition (ZOIs) of glycyl–histidine and methionyl–glycine dipeptides against *C. albicans* ATCC 10231, *E. coli* ATCC 35218, *K. pneumoniae* MTCC 109, *P. aeruginosa* ATCC 27853, and *S. aureus* ATCC 25923 were determined to evaluate their antimicrobial activity. A penicillin–streptomycin–amphotericin B solution (Biological Industries, Beit HaEmek, Israel) was used as a reference control due to its broad-spectrum antimicrobial activity against both bacterial and fungal microorganisms, providing a suitable comparative standard for the tested strains.

#### 3.4.1. Determination of Minimum Inhibitory Concentrations (MICs) and Minimum Microbicidal Concentrations (MMCs)

The minimum inhibitory concentrations (MICs) and minimum microbicidal concentrations (MMCs) of tested dipeptides were determined using the broth microdilution method. Briefly, each dipeptide was serially two-fold diluted in 96-well microtiter plates containing 100 µL of Brain Heart Infusion (BHI) broth per well. Subsequently, 10 µL of the standardized microbial suspension (adjusted to McFarland standard 2) belonging to each tested strain was added to each well containing decreasing concentrations of the dipeptides. The plates were incubated at 37 °C for 24 h, after which microbial growth was visually evaluated. The lowest concentration showing no visible growth was recorded as the MIC. To determine the MMC, 10 µL aliquots were taken from the wells of the MIC test and inoculated onto BHI agar plates without any dipeptides, which were then incubated at 37 °C for 24 h. The lowest concentration showing no visible growth on the agar surface was recorded as the MMC. All tests were performed in triplicate, and the median values were reported.

#### 3.4.2. Determination of Zones of Inhibition (ZOIs)

The inhibition zone diameters of the tested dipeptides were determined using the disk diffusion method. Briefly, sterile disks impregnated with 5 µL of 200 mg/mL glycyl–histidine and 5 µL of 200 mg/mL methionyl–glycine dipeptide solutions were placed on BHI agar plates previously inoculated with 100 µL of microbial suspension adjusted to McFarland standard 2. The plates were incubated at 37 °C for 24 h. After incubation, inhibition zones were measured using a ruler. All disk diffusion assays were conducted in triplicate, and the mean inhibition zone diameters were calculated.

### 3.5. Biofilm Inhibition

Biofilm inhibition of the tested isolates in BHI medium supplemented with glycyl–histidine and methionyl–glycine dipeptides was evaluated using the crystal violet assay [13]. Briefly, 10 µL of standardized microbial suspensions were inoculated into 96-well plates containing 100 µL of BHI with dipeptides at sub-MICs. Wells with BHI containing sub-MICs of penicillin–streptomycin–amphotericin B served as controls. After 24 h of incubation at 37 °C, non-adherent cells were removed by washing, and wells were stained with 1% (*w*/*v*) crystal violet for 25 min. Stained biofilms were rinsed, solubilized with 96% (*v*/*v*) ethanol for 25 min, and absorbance was measured at 560 nm. Biofilm formation in untreated wells was defined as 100%, and inhibition (%) was calculated relative to this positive control using the following formula (Equation (1)):

%Decrease = [(Absorbance Control (560 nm) − Absorbance Treatment (560 nm))/Absorbance Control (560 nm)] × 100(1)

In addition to quantitative biofilm biomass measurement using the crystal violet assay, light microscopy was employed to qualitatively support and visualize the effects of dipeptide treatment on biofilm formation.

### 3.6. Computational Details

Optimized structures of the investigated dipeptides were calculated using the Gaussian 09 W package and Gauss View 5.0 interface programs, respectively. In silico molecule docking calculations were performed with AutoDock Vina v1.2.7 (The Scripps Research Institute, La Jolla, CA, USA). In the final section, the SwissADME platform (http://www.swissadme.ch/) was used via SMILE codes to obtain drug similarity and various ADMET properties.

## 4. Conclusions

In conclusion, this study identifies glycyl–histidine as a particularly promising dipeptide, exhibiting both antimicrobial and antibiofilm efficacy against clinically relevant bacterial pathogens and *C. albicans*. This has also been confirmed by docking-supported studies. In contrast to methionyl–glycine, the consistent performance of glycyl–histidine across different microorganisms highlights the functional importance of its histidine residue and supports its potential as a building block for the development of novel antimicrobial and antibiofilm strategies, thereby providing guidance for future studies aimed at controlling biofilm-associated persistent infections.

## Figures and Tables

**Table 1:** MICs and MMCs of glycyl–histidine and methionyl–glycine dipeptides and the reference antibiotic solution (penicillin–streptomycin–amphotericin B) against *Candida albicans* ATCC 10231, *Escherichia coli* ATCC 35218, *Klebsiella pneumoniae* MTCC 109, *Pseudomonas aeruginosa* ATCC 27853, and *Staphylococcus aureus* ATCC 25923.

	Glycyl– Histidine (mg/mL)		Methionyl– Glycine (mg/mL)		Penicillin–Streptomycin–Amphotericin B Solution * (μL/mL)	
	MIC	MMC	MIC	MMC	MIC	MMC
*C. albicans* ATCC 10231	50	>50	>100	>100	>500	>500
*E. coli* ATCC 35218	12.5	>12.5	≥100	≥100	3.9	15.6
*K. pneumoniae* MTCC 109	12.5	>12.5	≥100	≥100	62.5	125
*P. aeruginosa* ATCC 27853	12.5	12.5	≥100	≥100	7.8	15.6
*S. aureus* ATCC 25923	12.5	>12.5	≥100	≥100	15.6	15.6

* Penicillin–streptomycin–amphotericin B solution: penicillin G, 10,000 U/mL; streptomycin, 10 mg/mL; amphotericin B, 25 µg/mL.

**Table 2:** Disk diffusion results of glycyl–histidine and methionyl–glycine dipeptides and reference antibiotic disks (amphotericin B, 100 µg; imipenem, 10 µg) against *C. albicans* ATCC 10231, *E. coli* ATCC 35218, *K. pneumoniae* MTCC 109, *P. aeruginosa* ATCC 27853, and *S. aureus* ATCC 25923.

	Glycyl–Histidine (200 mg/mL)	Methionyl–Glycine (200 mg/mL)	Amfotericin B (100 μg)	Imipenem (10 μg)
*C. albicans* ATCC 10231	– *	– *	16.0 ± 1.41	– *
*E. coli* ATCC 35218	8.3 ± 0.58	– *	– *	29.5 ± 2.12
*K. pneumoniae* MTCC 109	8.7 ± 0.58	– *	– *	36.5 ± 0.71
*P. aeruginosa* ATCC 27853	9.0 ± 1.0	– *	– *	19.5 ± 0.71
*S. aureus* ATCC 25923	8.3 ± 0.58	– *	– *	49.5 ± 0.71

* no inhibition zone detected. Amphotericin B (100 µg) disks were tested only against *C. albicans* ATCC 10231, whereas imipenem (10 µg) disks were evaluated against *E. coli* ATCC 35218, *K. pneumoniae* MTCC 109, *P. aeruginosa* ATCC 27853, and *S. aureus* ATCC 25923. Values represent mean ± SD of three independent experiments. Inhibition zone diameters are given in millimeters (mm).

**Table 3:** Docking results of dipeptides against 2UVO and 8BN6 receptors.

Mode	GLYH + PDB: 2UVO			MGLY + PDB: 2UVO		
	Affinity (kcal/mol)	RMSD l.b. (Å)	RMSD u.b. (Å)	Affinity (kcal/mol)	RMSD l.b. (Å)	RMSD u.b. (Å)
1	−6.8	0.000	0.000	−6.4	0.000	0.000
2	−5.8	1.799	2.377	−5.3	2.532	4.806
3	−4.6	31.847	33.222	−4.1	3.005	4.942
4	−4.6	14.240	16.360	−4.0	31.407	32.671
5	−4.5	14.357	16.301	−3.9	17.135	18.826
6	−4.5	15.494	17.044	−3.9	3.055	5.460
7	−4.5	20.783	22.321	−3.9	13.646	15.412
8	−4.4	15.277	17.336	−3.9	15.598	16.642
9	−4.4	15.099	16.923	−3.8	3.174	5.517
10	−4.4	14.433	15.442	−3.8	15.401	16.538
H.B = 8 Ki = 10.3649 μM				H.B = 5 Ki = 66.3544 μM		
Mode	GLYH + PDB: 8BN6			MGLY + PDB: 8BN6		
1	−5.7	0.000	0.000	−6.3	0.000	0.000
2	−5.5	1.640	4.077	−5.2	3.540	6.042
3	−5.5	1.820	5.143	−5.1	3.090	5.221
4	−5.1	2.746	4.553	−5.0	2.363	2.907
5	−5.1	13.406	14.869	−4.9	3.897	6.954
6	−5.1	1.749	2.173	−4.8	3.629	6.099
7	−5.1	17.911	19.708	−4.7	15.065	16.333
8	−5.0	3.227	5.525	−4.7	3.735	6.014
9	−4.9	2.859	3.827	−4.7	3.827	6.162
10	−4.9	2.578	3.705	−4.7	3.337	5.014
H.B = 5 Ki = 20.3594 μM				H.B = 0 Ki = 24.1027 μM		

**Table 4:** SwissADME predicted properties of dipeptides.

Category	Parameter	Glycyl–Histidine Values	Methionyl–Glycine Values
Physicochemical	Molecular formula	C_8_H_12_N_4_O_3_	C_7_H_14_N_2_O_3_S
	Molecular weight	212.21	206.26
	Heavy atoms	15	13
	Aromatic heavy atoms	0	0
	Fraction Csp^3^	0.50	0.71
	Rotatable bonds	6	7
	H-bond acceptors	6	4
	H-bond donors	3	3
	Molar refractivity	59.96	50.83
	TPSA	117.14	117.72
Lipophilicity	Log P_o_/𝓌 (iLOGP)	0.08	0.59
	Log P_o_/𝓌 (XLOGP3)	−4.38	−3.01
	Log P_o_/𝓌 (WLOGP)	−2.38	−0.73
	Log P_o_/𝓌 (MLOGP)	−1.68	−0.64
	Log P_o_/𝓌 (SILICOS-IT)	−0.22	−0.43
	Consensus Log P_o_/𝓌	−1.71	−0.84
Water solubility	Log S (ESOL)	2.00	1.24
	Solubility	2.12 × 10^4^ mg/mL	3.58 × 10^3^ mg/mL
	Class	Highly soluble	Highly soluble
	Log S (Ali)	2.53	1.10
	Solubility	7.26 × 10^4^ mg/mL	2.60 × 10^3^ mg/mL
	Class	Highly soluble	Highly soluble
	Log S (SILICOS-IT)	0.01	−0.66
	Solubility	2.18 × 10^2^ mg/mL	4.48 × 10^1^ mg/mL
	Class	Soluble	Soluble
Pharmacokinetics	GI absorption	Low	High
	BBB permeant	No	No
	P-gp substrate	No	No
	CYP1A2 inhibitor	No	No
	CYP2C19 inhibitor	No	No
	CYP2C9 inhibitor	No	No
	CYP2D6 inhibitor	No	No
	CYP3A4 inhibitor	No	No
	Log Kp (skin permeation)	−10.70	−9.70
Drug-likeness	Lipinski	Yes; 0 violations	Yes; 0 violations
	Ghose	No; 1 violation (WLOGP < −0.4)	No; 1 violation (WLOGP < −0.4)
	Veber	Yes	Yes
	Egan	Yes	Yes
	Muegge	No; 1 violation (XLOGP3 < −2)	No; 1 violation (XLOGP3 < −2)
	Bioavailability score	0.55	0.55
Medicinal chemistry	PAINS	0 alert	0 alert
	Brenk	0 alert	0 alert
	Lead-likeness	No; 1 violation (MW < 250)	No; 1 violation (MW < 250)

## Data Availability

The datasets generated and/or analyzed during the current study are available from the corresponding author upon reasonable request.

## Abbreviations

The following abbreviations are used in this manuscript:

2UVO	Protein Data Bank (PDB) ID of the antibiofilm target protein
8BN6	Protein Data Bank (PDB) ID of the antimicrobial target protein
ADME	Absorption, Distribution, Metabolism, and Excretion
ADMET	Absorption, Distribution, Metabolism, Excretion, and Toxicity
AMP	Antimicrobial Peptide
ATCC	American Type Culture Collection
BHI	Brain Heart Infusion
DFT	Density Functional Theory
ESKAPE	*Enterococcus faecium*, *Staphylococcus aureus*, *Klebsiella pneumoniae*, *Acinetobacter baumannii*, *Pseudomonas aeruginosa*, and *Enterobacter* species
GLYH	Glycyl–Histidine
LPS	Lipopolysaccharide
MGLY	Methionyl–Glycine
MIC	Minimum Inhibitory Concentration
MMC	Minimum Microbicidal Concentration
MTCC	Microbial Type Culture Collection
PDB	Protein Data Bank
RMSD	Root Mean Square Deviation
